# Longitudinal deformation models, spatial regularizations and learning strategies to quantify Alzheimer's disease progression

**DOI:** 10.1016/j.nicl.2014.02.002

**Published:** 2014-04-01

**Authors:** Jean-Baptiste Fiot, Hugo Raguet, Laurent Risser, Laurent D. Cohen, Jurgen Fripp, François-Xavier Vialard, for the Alzheimer's Disease Neuroimaging Initiative

**Affiliations:** a IBM Research, Smarter Cities Technology Centre, Damastown, Dublin 15, Ireland; b CEREMADE, UMR 7534 CNRS, Université Paris Dauphine, PSL★, France; c CSIRO Preventative Health National Research Flagship ICTC, The Australian e-Health Research Centre — BioMedIA, Royal Brisbane and Women's Hospital, Herston, QLD, Australia; d CNRS, Institut de Mathématiques de Toulouse, UMR 5219, France

**Keywords:** Alzheimer's disease, Brain imaging, Deformation model, LDDMM, Disease progression, Karcher mean, Transport, Logistic regression, Spatial regularization, Coefficient map

## Abstract

In the context of Alzheimer's disease, two challenging issues are (1) the characterization of local hippocampal shape changes specific to disease progression and (2) the identification of mild-cognitive impairment patients likely to convert. In the literature, (1) is usually solved first to detect areas potentially related to the disease. These areas are then considered as an input to solve (2). As an alternative to this sequential strategy, we investigate the use of a classification model using logistic regression to address both issues (1) and (2) simultaneously. The classification of the patients therefore does not require any a priori definition of the most representative hippocampal areas potentially related to the disease, as they are automatically detected. We first quantify deformations of patients' hippocampi between two time points using the *large deformations by diffeomorphisms* framework and transport these deformations to a common template. Since the deformations are expected to be spatially structured, we perform classification combining logistic loss and *spatial regularization* techniques, which have not been explored so far in this context, as far as we know. The main contribution of this paper is the comparison of regularization techniques enforcing the coefficient maps to be spatially smooth (Sobolev), piecewise constant (total variation) or sparse (fused LASSO) with standard regularization techniques which do not take into account the spatial structure (LASSO, ridge and ElasticNet). On a dataset of 103 patients out of ADNI, the techniques using spatial regularizations lead to the best classification rates. They also find coherent areas related to the disease progression.

## Highlights

• Study of deformation models for longitudinal analysis
• New framework combining LDDMM, logistic regression and spatial regularizations
• Simultaneous disease progression classification and biomarker identification
• Validation in the context of Alzheimer's disease on a large dataset from ADNI

## 1 Introduction

Large scale population studies aim to improve the understanding of the causes of diseases, define biomarkers for early diagnosis, and develop preventive treatments. An important challenge for medical imaging is to analyze the variability in MRI acquisitions of normal control (NC), mild cognitive impairment (MCI), and Alzheimer's disease (AD) patients. For Alzheimer's disease, several classification strategies have been proposed to separate patients according to their diagnosis. These methods can be split into three categories: voxel-based (Fan et al., 2007, 2008a,b; Klöppel et al., 2008; Lao et al., 2004; Magnin et al., 2009; Vemuri et al., 2008), cortical-thickness-based (Desikan et al., 2009; Klöppel et al., 2008; Querbes et al., 2009) and hippocampus-based (Chupin et al., 2007, 2009; Gerardin et al., 2009) methods. While decent classification rates can be achieved to separate AD from NC or NC from p-MCI (progressive MCI patients, i.e. converting to AD), all methods perform poorly at separating s-MCI (stable MCI patients, i.e. non-converting to AD) and p-MCI. A recent review comparing these methods can be found in Cuingnet et al. (2011).

In the case of longitudinal analysis, it is not anymore the shapes that are compared but their evolutions in time. To extract information between two successive time-points, we use a one-to-one deformation which maps the first image onto the second one. Different registration algorithms are available to compute plausible deformations in this context. However, only one, the *large deformations via diffeomorphisms (LDDMM)* (Beg et al., 2005), provides a Riemannian setting that enables to represent the deformations using tangent vectors: initial velocity fields or equivalently initial momenta. This can be used in practice to retrieve local information and to perform statistics on it as presented in Vaillant et al. (2004) and Wang et al. (2007). In this direction, it is worth mentioning the study of Singh et al. (2010) which shows the correlation between principal modes of deformation and diagnosis. In Qiu et al. (2008) the authors estimate the typical deformation of several clinical groups from the deformations between baseline and follow-up hippocampus surfaces. In order to compare this information across the population, we need to define a common coordinate system. This implies (1) the definition of a template and (2) a methodology for the transport of the tangent vector information. Note finally that, as far as the authors know, no paper explores binary classification using logistic regression in this context.

Quality of shape descriptors with regard to the disease is often evaluated through statistical significance tests or classification performance. In this paper, we evaluate descriptors on a binary classification task using logistic regression.

In addition to its simplicity, it has the advantage of providing a map of coefficients weighting the relevance of each voxel. Such map can be used to localize the hippocampus deformations that are related to AD. However, the dimensionality of the problem (i.e. number of voxels *p*) being much higher than the number of observations (i.e. number of patients *n*, p ~ 10^6^ ≫ *n* ~ 10^2^), the problem requires proper regularization.

Now standard regularization methods such as *ridge* (Hoerl and Kennard, 1970), *LASSO* (Tibshirani, 1994) and *Elastic Net* (Zou and Hastie, 2005) do not take into account any spatial structure of the coefficients.

In contrast, spatial models for regularizing supervised learning methods have been proposed in the literature (Grosenick et al., 2013; Jenatton et al., 2012; Ng and Abugharbieh, 2011). *Total variation* was used to regularize a logistic regression on functional MRI (fMRI) data (Michel et al., 2011). This method promotes coefficient maps with spatially homogeneous clusters. *Fused LASSO* was also used on fMRI data (Baldassarre et al., 2012; Gramfort et al., 2013). Similar ideas can be found in Cuingnet et al. (2012) where the authors defined the notion of spatial proximity to regularize a linear SVM classifier.

In Durrleman et al. (2013), the authors introduce sparse parametrization of the diffeomorphisms in the LDDMM framework. Our goal is different: we want spatial properties (smoothness, sparsity, etc.) to be found *across* the population (i.e. on the common template) and we want this coherence to be driven by the disease progression.

In this paper, we investigate the use of total variation, Sobolev and fused LASSO regularizations in 3D volumes. Compared to total variation, Sobolev enforces smoothness of the coefficient map, whereas fused LASSO adds a sparsity constraint.

The deformation model used to assess longitudinal evolutions in the population is presented in Section 2. Machine learning strategies are discussed and the model of classification with logistic loss and spatial regularization is described in Section 3. The dataset used and numerical results are presented in Section 4. We illustrate that initial momenta capture information related to AD progression, and that spatial regularizations significantly increase classification performance. Section 5 concludes the paper.

## 2 Longitudinal deformation model for population analysis

### 2.1 Global pipeline

Let us assume that we have a population of patients and the binary segmentation of their hippocampus at two different time points, called *screening* and *follow-up*. Let us also assume that all patients have the same diagnosis at the screening time point, and only a part of them have converted to another diagnosis at the follow-up time point. Our goal is to compare patient evolutions, and classify them with regard to disease progression, i.e. stable diagnosis versus progressive diagnosis. From a machine learning point of view, we need to build features encoding the evolutions of the patients.

**Fig. 1:**
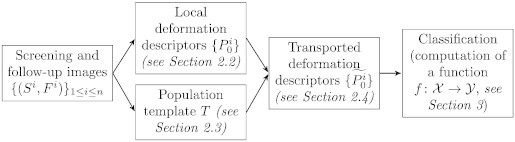
Four steps are needed to classify patient evolutions using local descriptors of shape deformations: (1) the local descriptors are computed for each patient independently, (2) a population template is computed, (3) all local shape deformation descriptors are transported towards this template, and (4) classification is performed.

We use the pipeline summarized in Fig. 1. First, the evolution descriptors are computed locally for each patient (independently). To be able to compare these descriptors, one needs to transport them into a common space. To do so, a population template is computed, towards which all the local descriptors are transported. Finally, classification is performed to separate progressive from stable patients.

### 2.2 Diffeomorphic registration via geodesic shooting

As mentioned in Sections 1 and 2.1, local deformation descriptors are computed to model the evolutions of the patients. In this section, we describe how we use diffeomorphic registration via geodesic shooting Vialard et al. (2012a) to compute these local deformation descriptors.

#### 2.2.1 Definitions

To register a source image *I* : *Ω* ⊂ ℝ^3^ → ℝ towards a target image *J* : *Ω* ⊂ ℝ^3^ → ℝ, the LDDMM framework (Beg et al., 2005) introduces the following minimization problem

(1) $$\underset{\upsilon \in L^{2}\left(\left[0,1\right],H_{K}\right)}{\mathrm{argmin}}\frac{1}{2}∥I\circ \phi_{0,1}^{-1}-J{∥}_{L^{2}}^{2}+\lambda \int_{0}^{1}∥\upsilon_{t}{∥}_{K}^{2}\mathrm{dt},$$

where *υ* : (*t*,*ω*) ∈ [0,1] × *Ω* ⊂ ℝ^3^ → *Ω* is a time dependent velocity field that belongs to a reproducing kernel Hilbert space $H_{K}$ of smooth enough vector fields defined on Ω, and of associated kernel *K* and norm ∥ ∥_*K*_, and *λ* ≥ 0 is a regularization coefficient. For (*t*,*ω*) ∈ [0,1] × *Ω*, we note *υ_t_*(ω) = *υ*(*t*, ω). The deformation *ϕ* : [0,1]^2^ × *Ω* ⊂ ℝ^3^ → *Ω* is given by the flow of *υ_t_*

(2) $$\forall \left(t,\omega \right)\in \left[0,1\right]\times \Omega ,\;\left\{\begin{array}{l} \frac{\partial \phi_{0,t}}{\partial t}\left(\omega \right)=\upsilon_{t}\circ \phi_{0,t}(\omega )\\ \phi_{t,t}\left(\omega \right)=\omega \;, \end{array}\right)$$

where *ϕ*_*t*1, *t*2_ is the deformation from *t* = *t*_1_ to *t* = *t*_2_. Such approach induces a Riemannian metric on the orbit of *I*, i.e. the set of all deformed images by the registration algorithm (Miller et al., 2006). The first term in formula (1) is a similarity term controlling the matching quality whereas the second one is a smoothing term controlling the deformation regularity. Now noting $I_{t}\overset{\mathrm{def}.}{=}\;I\circ \phi_{0,t}^{-1}$ and $J_{t}\overset{\mathrm{def}.}{=}\;J\circ \phi_{t,1}$, the Euler-Lagrange equation associated with Eq. (1) reads ∀ (*t*,*ω*) ∈ [0,1] × *Ω*,

(3) $$\upsilon_{t}\left(\omega \right)=-K⋆\left(\mathrm{grad}I_{t}\left(\omega \right){\mathrm{Jac}}_{\phi_{t,1}}\left(\omega \right)\left(I_{t}\left(\omega \right)-J_{t}\left(\omega \right)\right))\right),$$

where *K* is the translation-invariant kernel of the reproducing kernel Hilbert space, ⋆ the convolution operator, grad the image gradient in space and Jac_*ϕ*_ the Jacobian of *ϕ*.

For *t* ∈ [0,1], let us define the momentum *P*_*t*_ : *Ω* → ℝ by

(4) $$\forall \omega \in \Omega ,\;P_{t}\left(\omega \right)\overset{\mathrm{def}.}{=}\;{\mathrm{Jac}}_{\phi_{t,1}}\left(\omega \right)\left(I_{t}\left(\omega \right)-J_{t}\left(\omega \right)\right).$$

The Euler–Lagrange Eq. (3) can be rewritten as a set of geodesic shooting equations

(5) $$\forall \left(t,\omega \right)\in \left[0,1\right]\times \Omega ,\;\left\{\begin{array}{l} \frac{\partial I_{t}}{\partial t}\left(\omega \right)+\langle \mathrm{grad}I(\omega ),\upsilon_{t}(\omega )\rangle =0,\\ \frac{\partial P_{t}}{\partial t}\left(\omega \right)+\mathrm{div}\left(P_{t}\left(\omega \right)\upsilon_{t}\left(\omega \right)\right)=0,\\ \upsilon_{t}\left(\omega \right)+K⋆\mathrm{grad}I_{t}(\omega )\;P_{t}(\omega )=0, \end{array}\right)$$

where div is the divergence operator.

Given an initial image *I*_0_ and an initial momentum *P*_0_, one can integrate the system (Eq. (5)). Such a resolution is called *geodesic shooting*. We say that *we shoot from I*_0_ *using P*_0_.

The minimization problem (Eq. (1)) can be reformulated using a shooting formulation on the initial momentum *P*_0_

(6) $$\underset{P_{0}}{\mathrm{argmin}}\frac{1}{2}∥I\circ \phi_{0,1}^{-1}-J{∥}_{L^{2}}^{2}+\lambda {\langle \mathrm{grad}I_{0}P_{0},K⋆\mathrm{grad}I_{0}P_{0}\rangle }_{L^{2}}\;$$

subject to the shooting system (Eq. (5)).

In order to solve the new optimization problem (Eq. (6)), we use the methodology described in Risser et al. (2011) and Vialard et al. (2012a). Note that this methodology is similar to the one presented in Ashburner and Friston (2011), but uses a different optimization strategy.

**Fig. 2:**
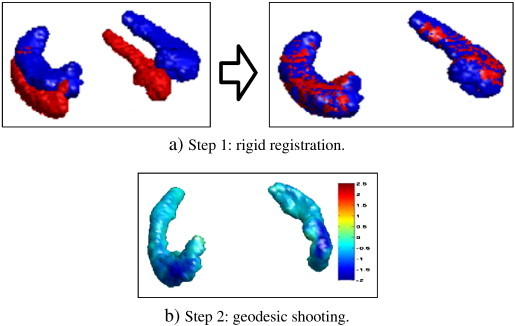
For each patient, the initial momentum encoding the hippocampus evolution is computed in a two-step process.

For each patient, a two-step process was performed to encode the deformations of the hippocampus shape evolution from the screening image *S* (scanned at *t* = *t_0_*) to the follow-up image *F* (scanned at *t* = *t_0_* + 12 months), as described in Fig. 2. First *F* was rigidly registered back to *S*. We note *R* : *Ω* ⊂ ℝ^3^ → *Ω* the rigid transformation obtained. Second, the geodesic shooting was performed with the screening image as source image (*I* = *S*) point towards the registered followed-up image as target image (*J* = *F* ∘ *R*^− 1^). Initial momenta from different patients are local descriptors that were used to compare hippocampus evolutions, such choice is further described in the next paragraph.

#### 2.2.2 Motivation and rationales for the use of initial momenta

As written in the third row of Eq. (5), the velocity field *υ* encoding the geodesic between the registered images has the following property at each time *t*∈ [0,1] and at each coordinate *ω* ∈ *Ω*,

(7) $$\upsilon_{t}\left(\omega \right)=-K⋆\mathrm{grad}I_{t}\left(\omega \right)P_{t}\left(\omega \right),$$

We recall that *I_t_*, *υ_t_* and *P_t_* are respectively the deformed source image, the velocity field and the momentum at time *t*. We also denote *K*⋆ the convolution with the kernel *K* (typically Gaussian). Therefore, Eq. (7) can be read in the case of a binary image as follows: the unitary vector field normal to the shape surface is multiplied by a scalar field *P*(*t*) and this quantity gives the vector field *υ_t_* once convolved with the kernel *K*.

The system given in all rows of Eq. (5) leads to the fact that the initial momentum *P*_0_ entirely controls the deformation for a given source image *I*_0_ and a given kernel *K*. In the context of our study, longitudinal variations of the geodesics are relatively limited as only small deformations are required to register pairs of hippocampi out of the same subject. The displacement field can then be reasonably approximated by *Id* + *υ*_0_ using a first-order expansion of Eq. (5). As a consequence, *P*_0_ can be directly interpreted as a value encoding expansions and contractions of the shape if multiplied by − grad *I*_0_ and then smoothed by *K*. Note also that the momentum is a scalar field, which is a more compact representation than a vector field. This motivates our approach.

### 2.3 Population template

#### 2.3.1 Need for a template

As mentioned in Section 2.1, local descriptors of hippocampus evolutions need to be transported in a common space prior to any statistical analysis. One way to obtain spatial correspondences between local descriptors of different patients consists in building a population template and then aligning these descriptors on the template. In the literature, template algorithms can be categorized into deterministic (Avants and Gee, 2004; Beg and Khan, 2006; Fletcher et al., 2004; Pennec, 2006; Vialard et al., 2011), probabilistic (Allassonnière et al., 2008; Ma et al., 2008) and mixed (Bhatia et al., 2004; Jia et al., 2010; Joshi et al., 2004; Seghers et al., 2004) approaches. As described in Section 4.1, we want to build a population of binary images of hippocampi. As there is no variation of topology, and we want a template with sharp boundaries without averaging the gray levels, the first category is appropriate. Most algorithms in this category rely on the notions of Fréchet and Karcher means, which we will now describe.

#### 2.3.2 Notions of Fréchet and Karcher means

In the Riemannian framework used for the geodesic shooting, a Fréchet mean (Fréchet, 1948) can be used to define an average shape from a population (Fletcher et al., 2004; Pennec, 1999, 2006). Given *n* images {*S*^*i*^ : *Ω* ⊂ ℝ^3^ → ℝ}_1 ≤ *i* ≤ *n*_ and *d* a Riemannian metric on the space of images, the *Fréchet mean* $\hat{T}:\Omega \subset {\mathbb{R}}^{3}\to \mathbb{R}$ is defined as a minimizer of the sum of the geodesic distances to all images

(8) $$\min_{T}\;\frac{1}{n}\sum_{i=1}^{n}\;d{\left(T,S^{i}\right)}^{2}.$$

In practice, such problem is often solved via an optimization procedure looking for a local minimum, and the solutions found are called *Karcher means*. For instance, a solution of Eq. (8) can be computed using a gradient descent procedure (Vialard et al., 2011).

#### 2.3.3 Invariance to rigid orientations, approximations and optimization procedure

The problem (8) is not invariant with respect to the rigid orientations of the input images, we modify the optimization problem to

(9) $$\min_{T,R^{1},\ldots ,R^{n}}\;\frac{1}{n}\sum_{i=1}^{n}\;d{\left(T,S^{i}\circ {\left(R^{i}\right)}^{-1}\right)}^{2}\;,$$

where {*R*^*i*^ : *Ω* → *Ω*}_1 ≤ *i* ≤ *n*_ are rigid transformations. In this paper, we assume that the solution of Eq. (9) can be approximated by alternate minimization. It is also important to note that in the general case there is not necessarily unicity of the solution.

When the {*R^i^*} are fixed, we follow the optimization strategy described in Vialard et al. (2011). Since the functional in Eq. (1) does not give a geodesic distance between two images — but between a source image and the deformed image, we approximate the minimization with regard to *T* by

(10) $$\min_{T}\;\frac{1}{n}\sum_{i=1}^{n}\;d{\left(T,J_{1}^{i}\right)}^{2},$$

where *J*_1_^*i*^ is the result of the shooting equations for the initial conditions *I* = *T* and *P*_0_ = *P*_0_^*i*^, where *P*_0_^*i*^ is a minimizer of Eq. (6) with *J* = *S*^*i*^ ∘ (*R*^*i*^)^1^. In this case, each term of the sum in Eq. (10) is equal to $<\mathrm{grad}IP_{0},K⋆\left(\mathrm{grad}IP_{0}\right){>}_{L^{2}}$, and the gradient with regard to *T* is

(11) $$-\frac{1}{n}\sum_{i=1}^{n}\;K⋆\mathrm{grad}TP_{0}^{i},$$

where *P*_0_^*i*^ is the initial momentum matching *T* on *S*^*i*^ ∘ (*R*^*i*^)^− 1^ via the shooting system (Eq. (5)).

When *T* is fixed, we approximate the optimization over {*R*^*i*^}_1 ≤ *i* ≤ *n*_ by performing rigid registrations matching each *S^i^* to *T*.

Altogether, each update of the Karcher estimate is composed of four steps

1. the images *S^i^* are rigidly aligned towards the current Karcher mean estimate *T_k_*,
2. diffeomorphic registrations via geodesic shootings from the current Karcher estimate *T_k_* towards all the registered images *S*^*i*^ ∘ (*R*^*i*^)^− 1^ are computed,
3. geodesic shooting from *T_k_* using $P_{0}^{\mathrm{mean}}\overset{\mathrm{def}.}{=}\;\frac{1}{n}\sum_{i}\;P_{0}^{i}$ generates a deformation field *u_mean_*,
4. the composed deformation field $u_{k+1}\overset{\mathrm{def}.}{=}\;u_{\mathrm{mean}}\circ u_{k}$ is used to compute the updated estimate from the reference image.

The advantage of computing the new estimate from a reference image is to avoid consecutive resamplings that would lead to smoothing and bias, as noted in Yushkevich et al. (2010).

In the literature, the empirical convergence of the gradient descent procedure optimizing over *T* (with {*R*_*i*_}_1 ≤ *i* ≤ *n*_ fixed) was studied in Vialard et al. (2011, 2012b). Similar tests are performed in Section 4.2 for our procedure.

### 2.4 Tangent information and associated transport

#### 2.4.1 Motivation and rationals

The local descriptors computed for each patient as explained in Section 2.2 need to be transported in a common coordinate space: the space of the Karcher average defined in Section 2.3.

There is still no consensus about the choice of which transport method should be used in our context. Different methods have been proposed. The first one is the transport of vector fields by the standard adjoint map. It was however shown that this method is not quite appropriate for statistical study (Bossa et al., 2010). Parallel transport was also proposed in the context of LDDMM (Younes, 2007). Although it might seem relevant in our context, volume variation may be distorted. Note that its properties also depend on the deformation path and not only on the final deformation.

In the context of LDDMM, another action of the group of deformations on the momentum is called co-adjoint transport (Fiot et al., 2012). This method only depends on the final deformation and preserves volume variation in the context of small deformations on binary images. This argument motivated its use in our study.

#### 2.4.2 Definitions

**Fig. 3:**
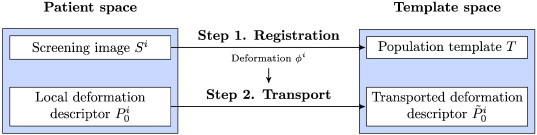
Local descriptors of hippocampus evolutions are transported to the template in a two-step process. First the deformation field from the patient space to the population template. Second, this deformation field is used to transport the local descriptors.

A two-step process was then used to transport local descriptors of hippocampus evolutions to the template space (Fig. 3). First, the screening hippocampus *S^i^* was registered towards the template *T* rigidly (Ourselin et al., 2001) then non-rigidly (Modat et al., 2010). The resulting deformation is denoted by *ϕ^i^*. Second, this transformation was used to transport the local descriptors of hippocampus deformations towards the template.

We use the standard transport for a density *P*_0_^*i*^ : *Ω* ⊂ ℝ^3^ → ℝ, defined by

(12) $$\forall \omega \in \Omega ,\;{\tilde{P}}_{0}^{i}\left(\omega \right)\overset{\mathrm{def}.}{=}\det\left({\mathrm{Jac}}_{{\left(\phi^{i}\right)}^{-1}}\left(\omega \right)\right)P_{0}^{i}\circ {\left(\phi^{i}\right)}^{-1}\left(\omega \right),$$

where det is the notation for the determinant. Note that this action preserves the global integration of the density by a simple change of variable.

## 3 Machine learning strategies

### 3.1 Support vector machine classification

In Fiot et al. (2012), SVM classifiers are used on different types of features. In that paper, local features obtained by integration of initial momenta on subregions provided the best classification results. This conclusion motivates the search for an optimal subregion Ω*_r_* defining features as $x_{i}\overset{\mathrm{def}.}{=}\int_{\Omega_{r}}{\tilde{P}}_{0}^{i}\left(\omega \right)\mathrm{d\omega}$ (optimal in terms of classification accuracy). This is equivalent to the search of the best indicator function *I*_*r*_ : *Ω* → {0,1}, or more generally a weighting function *w* : *Ω* → ℝ defining features by $x_{i}\overset{\mathrm{def}.}{=}\int_{\Omega }w\left(\omega \right){\tilde{P}}_{0}^{i}\left(\omega \right)\mathrm{d\omega}$.

To compute meaningful weighting functions, models where the *feature space* is the same as the *input space* are of particular interest. Indeed as one coefficient corresponds to one voxel, meaningful spatial regularizations can be introduced. This was used in the linear SVM setting in Cuingnet et al. (2012). In this paper, we exploit similar ideas on a classification framework with a logistic loss, which is well-suited for the introduction of spatial regularizations, easy to implement and that can be solved efficiently.

### 3.2 Binary classification with logistic regression and spatial regularization

#### 3.2.1 Definitions

Let us define a predictive model which reads

(13) $$y\overset{\mathrm{def}.}{=}F\left(\mathrm{Xw}+b\right),$$

where **y** ∈ {± 1}*^n^* is the behavioral variable, **X** ∈ ℝ^*n* × *p*^ is the design matrix containing *n* observations of dimension *p*, *F* is the prediction function and (**w**,*b*) ∈ ℝ^*p*^ × ℝ are the parameters to estimate. In our application, each coefficient in **y** represents the disease progression of one of the *n* patients, and each row in **X** contains the initial momentum representing the deformations of the hippocampus of one of the *n* patients. It is important to notice that each row in **X** is noted as a vector in ℝ^*p*^ in the formulation of the predictive model, but it is actually a scalar field in 3D. Similarly, **w** is noted as a vector in ℝ^*p*^ for the convenience of the formulation of the model, even if it also represents a scalar field in 3D. Since each coefficient in **w** is associated to a spatial position, **w** is sometimes called a *coefficient map*. Such property allows us to detect (spatial) areas of interest, with regard to the machine learning problem we want to solve (see Section 3.2.4 about the interpretation of the solution of the model).

The logistic regression model defines the probability of observing *y_i_* given the data **x***_i_* as

(14) $$p\left(y_{i}|x_{i},w,b\right)\overset{\mathrm{def}.}{=}\frac{1}{1+\exp\left(-y_{i}\left(x_{i}^{T}w+b\right)\right)}.$$

Given parameters $\left(\hat{w},\hat{b}\right)$ and a new data point **x** the prediction is the maximum likelihood, i.e. $\mathrm{class}\left(x\right)={\mathrm{argmax}}_{y\in \left\{\pm 1\right\}}p\left(y|x,\hat{w},\hat{b}\right)=\mathrm{sign}\left(x^{T}\hat{w}+\hat{b}\right)$. Accordingly the parameters are estimated as minimizers of the opposite log likelihood of the observations, considered as independent

(15) $$L\left(w,b\right)\overset{\mathrm{def}.}{=}\;\frac{1}{n}\sum_{i=1}^{n}\log\left(1+\exp\left(-y_{i}\left(x_{i}^{T}w+b\right)\right)\right).$$

Since the number of observations is much smaller than the dimension of the problem (*n* ≪ *p*) minimizing directly the loss Eq. (15) leads to overfitting, and proper regularization is required. This is commonly performed by introducing a regularization function *J* and the final problem becomes

(16) $$\mathrm{Find}\left(\hat{w},\hat{b}\right)\mathrm{in}\underset{w,b}{\mathrm{argmin}}L\left(w,b\right)+\lambda J\left(w\right),$$

where *λ* is a coefficient tuning the balance between loss and regularization.

The standard *elastic net* regularization (Zou and Hastie, 2005) uses a combined ℓ_1_ and squared ℓ_2_ penalization $\mathrm{\lambda EN}\left(w\right)\overset{\mathrm{def}.}{=}\lambda_{1}||w||{}_{1}+\lambda_{2}||w||{}_{2}^{2}=\sum_{j=1}^{p}\lambda_{1}|w_{j}|+\lambda_{2}w_{j}^{2}$, with the limit cases *λ*_2_ = 0 referred to as *LASSO* (Tibshirani, 1994) and *λ*_1_ = 0 referred to as *ridge* (Hoerl and Kennard, 1970). However as mentioned in Michel et al. (2011), one drawback of such methods is that they do not take into account any geometrical structure of **w**. Since coefficients are expected to be locally correlated in space, we investigate the Sobolev semi-norm, total variation semi-norm and fused-LASSO regularizations, respectively defined as

(17) $$\mathrm{SB}\left(w\right)\overset{\mathrm{def}.}{=}\sum_{\omega \in \Omega }∥{\mathrm{grad}}_{\Omega }w\left(\omega \right){∥}_{2}^{2},$$

(18) $$\mathrm{TV}\left(w\right)\overset{\mathrm{def}.}{=}\sum_{\omega \in \Omega }∥{\mathrm{grad}}_{\Omega }w\left(\omega \right){∥}_{2},$$

(19) $$\lambda \mathrm{FL}\left(w\right)\overset{\mathrm{def}.}{=}\lambda_{1}\mathrm{TV}\left(w\right)+\lambda_{2}∥w{∥}_{1}.$$

The above sums go over all voxels *ω* in the domain *Ω* ⊂ ℝ^3^, and grad_Ω_ is a linear operator implementing the image gradient by finite differences. By indexing each voxel *ω* by integer coordinates on a 3D lattice, we define grad_Ω_ by

(20) $${\mathrm{grad}}_{\Omega }w\left(\omega_{\mathrm{ijk}}\right)\overset{\mathrm{def}.}{=}\left(\begin{array}{l} \Delta_{\Omega }w\left(\omega_{\mathrm{ijk}},\omega_{\left(i+1\right)\mathrm{jk}}\right)\\ \Delta_{\Omega }w\left(\omega_{\mathrm{ijk}},\omega_{i\left(j+1\right)k}\right)\\ \Delta_{\Omega }w\left(\omega_{\mathrm{ijk}},\omega_{\mathrm{ij}\left(k+1\right)}\right) \end{array}\right),$$

where $\Delta_{\Omega }w\left(\omega_{1},\omega_{2}\right)\overset{\mathrm{def}.}{=}\left\{\begin{array}{ll} w\left(\omega_{2}\right)-w(\omega_{1}) & \mathrm{if}\left(\omega_{1},\omega_{2}\right)\in \Omega^{2},\\ 0 & \mathrm{otherwise}. \end{array}\right)$ This definition allows to restrain Ω to any region of interest and boundaries of the domain are not penalized. Rationals and differences for those regularizations are discussed in Section 4.

#### 3.2.2 Solving the model

Let us first study differentiability and convexity of the objective function in Eq. (15). For convenience, we define $\tilde{w}\overset{\mathrm{def}.}{=}\;{\left(w^{T},b\right)}^{T}$ and for all *i*, ${\tilde{x}}_{i}\overset{\mathrm{def}.}{=}\;{\left(x_{i}^{T},1\right)}^{T}$, with associated data matrix $\tilde{X}\overset{\mathrm{def}.}{=}{\left({\tilde{x}}_{\mathrm{ij}}\right)}_{\begin{array}{l} 1\leq i\leq n\\ 1\leq j\leq p+1 \end{array}}\in {\mathbb{R}}^{n\times \left(p+1\right)}$. Then Eq. (15) becomes

(21) $$L\left(\tilde{w}\right)=\frac{1}{n}\sum_{i=1}^{n}\log\left(1+\exp\left(-y_{i}{\tilde{x}}_{i}^{T}\tilde{w}\right)\right).$$

This loss function is twice differentiable and the non-negativity of $\nabla^{2}L\left(\tilde{w}\right)$ establishes the convexity.

When the regularization *J* is also convex and twice differentiable the reference optimization algorithms include quasi-Newton methods; in particular for large-scale problems the limited memory Broyden–Fletcher–Goldfarb–Shanno (LM-BFGS) is very popular. However non-differentiable regularizations such as total variation and fused LASSO optimization raises theoretical difficulties. Proximal methods such as monotonous fast iterative shrinkage thresholding algorithm (M-FISTA, (Beck and Teboulle, 2009)) and generalized forward–backward (GFB, (Raguet et al., 2013)) have been considered. Unfortunately their low convergence rates are prohibitive for extensive investigation of the classification scheme (parameter *λ*, domain Ω, training design matrix **X**). Therefore we used the hybrid algorithm for non-smooth optimization (HANSO, (Lewis and Overton, 2012)) which is a LM-BFGS algorithm with weak Wolfe conditions line search. This addresses both the total variation semi-norm and the ℓ_1_-norm, with almost everywhere

$$\begin{array}{l} \nabla \mathrm{TV}\left(w\right)=-\mathrm{div}\left({\left({\left\|{\mathrm{grad}}_{\Omega }w\left(\omega \right)\right\|}_{2}^{-1}{\mathrm{grad}}_{\Omega }w\left(\omega \right)\right)}_{\omega \in \Omega }\right),\\ \nabla {\left\|w\right\|}_{1}={\left(\mathrm{sign}\left(w\left(\omega \right)\right)\right)}_{\omega \in \Omega }. \end{array}$$

#### 3.2.3 Weighted loss function

In supervised learning, classifiers trained with observations not equally distributed between classes can be biased in favor of the majority class. In order to alleviate this, several strategies can be used. One strategy is to restrict the training set to be equally distributed among classes. An alternative strategy is to use the full training set and introduce weights (*q*_*i*_)_*i* ∈ [[1,*n*]]_ in the loss function as follows

(22) $$L_{q}\left(\tilde{w}\right)\overset{\mathrm{def}.}{=}\frac{1}{n}\sum_{i=1}^{n}q_{i}\log\left(1+\exp\left(-y_{i}{\tilde{x}}^{T}\tilde{w}\right)\right)$$

where $q_{i}\overset{\mathrm{def}.}{=}n/\left(n_{c}\times \mathrm{card}\left\{j\in \left[1..n\right]|y_{j}=y_{i}\right\}\right)$, *n_c_* being the number of classes (2 in our case). When the observations are equally distributed among classes *q*_i_ = 1 for all *i* and one retrieves (Eq. (21)), whereas *q*_i_ < 1 (respectively *q*_i_ > 1) when the class of observation *i* is over-represented (respectively under-represented) in the training set.

#### 3.2.4 Interpretation of the solution

Another motivation for the use of the model presented in Section 3 is the possibility to interpret the computed solution. Let us remind that, after optimization, the solution is of the form $\left(\hat{w},\hat{b}\right)\in {\mathbb{R}}^{p}\times \mathbb{R}$. This solution can be used to predict the evolution *y*∈ {± 1} of a new patient of associated initial momentum **x** ∈ ℝ^*p*^, by using the equation $y=\mathrm{sign}\left(x^{T}\hat{w}+\hat{b}\right)$. As mentioned in Section 3.2.1, the hyperplane $\hat{w}$ has the same dimension of the initial momentum, and each coefficient is associated to one voxel.

Now let us talk about the interpretation of the weights in $\hat{w}$. High coefficients in $\hat{w}$ correspond to *areas of the hippocampus where the deformation is related to the disease progression*. They are not areas of high expansions or contractions, and therefore have a different interpretation than the coefficients in the initial momenta (see Section 2.2 for the interpretation of the coefficients of the initial momenta). On the contrary, coefficients close to zero in $\hat{w}$ represent areas where the values of **x** are not relevant to the disease progression (in that case the values of **x** in these areas will not modify the value of the scalar product $x^{T}\hat{w}$). In that sense, the coefficients in $\hat{w}$ have a clinical interpretation.

To summarize, each initial momentum can describe the local hippocampal shape changes *for a patient taken individually*, whereas the coefficient map $\hat{w}$ can describe the relevance of hippocampal areas with regard to the disease progression, *at the population level* i.e. from the observation of *all training patients*.

## 4 Material and results

### 4.1 Data

Data used in the preparation of this article were obtained from the Alzheimer's Disease Neuroimaging Initiative (ADNI) database (http://adni.loni.usc.edu). The ADNI was launched in 2003 by the National Institute on Aging, the National Instituteof Biomedical Imaging and Bioengineering, the Food and Drug Administration, private pharmaceutical companies and non-profit organizations, as a $60 million, 5-year public private partnership. The primary goal of ADNI has been to test whether serial MRI, positron emission tomography, other biological markers, and clinical and neuropsychological assessment can be combined to measure the progression of MCI and early AD. Determination of sensitive and specific markers of very early AD progression is intended to aid researchers and clinicians to develop new treatments and monitor their effectiveness, as well as lessen the time and cost of clinical trials.

The Principal Investigator of this initiative is Michael W. Weiner, MD, VA Medical Center and University of California — San Francisco. ADNI is the result of efforts of many co-investigators from a broad range of academic institutions and private corporations, and subjects have been recruited from over 50 sites across the U.S. and Canada. The initial goal of ADNI was to recruit 800 subjects but ADNI has been followed by ADNI-GO and ADNI-2. To date these three protocols have recruited over 1500 adults, ages 55 to 90, to participate in the research, consisting of cognitively normal older individuals, people with early or late MCI, and people with early AD. The follow-up duration of each group is specified in the protocols for ADNI-1, ADNI-2 and ADNI-GO. Subjects originally recruited for ADNI-1 and ADNI-GO had the option to be followed in ADNI-2. For up-to-date information, see http://www.adni-info.org.

A dataset of 206 hippocampus binary segmentations from 103 patients enrolled in ADNI (Mueller et al., 2005) has been used. The segmentations were computed and provided by ADNI, detailed information can be found on their website. For each patient, ‘screening’ and ‘month 12’ were the two time points selected. All patients were MCI at the screening point, 19 converted to AD by month 12, and the remaining 84 stayed MCI.

### 4.2 Experiments

#### 4.2.1 Preprocessing

First, all screening images were resampled to a common isotropic voxel size 1.0 × 1.0 × 1.0 mm, similar to their original size. Rigid transformations aligning the month 12 hippocampus towards the screening ones were computed using Ourselin et al. (2001).

#### 4.2.2 Computation of initial momenta

The geodesic shootings (Vialard et al., 2012a) were performed^3^ using a sum of three kernels (sizes 1, 3 and 6 mm, with respective weights 2, 1 and 1), and 200 gradient descent iterations. To check the quality of the geodesic shooting computed for each patient *i* (second step in Fig. 2), the evolution of the Dice score DSC between *S*_*t*_^*i*^ which is the deformed screening image at time *t* and the target image *F*^*i*^ ∘ (*R*^*i*^)^− 1^ was computed, and the average final DSC is 0.94 ± 0.01.

#### 4.2.3 Computation of the template

The computation of a Karcher mean as described in Section 2.3 is a computationally expensive step, which is linear with the number of images. Therefore it can be desirable to select only a subset of the images. In this paper, a subset of 20 images was used, of corresponding hippocampal volumes which were the closest to the mean hippocampal volume. The Karcher mean estimate was updated four times, with respectively 200, 150, 150 and 100 gradient descent iterations in the geodesic shootings. Below are two verifications we performed to validate this approach.

First, we evaluated if all patients can be registered properly to the template, which is an important verification since only a subset of the images was used to compute the template. In our study, the average Dice score between the 103 registered patients and the template was 0.87 ± 0.02, which validated the suitability of the template obtained for our study. The last paragraph of Section 4.3 also provides another reason why such template can be used in our study.

**Fig. 4:**
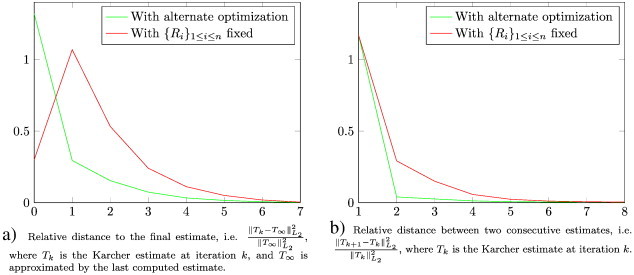
Empirical measures of convergence of the Karcher template algorithm. On this dataset, we notice that (1) the convergence speeds are coherent with the ones presented in Vialard et al. (2011) and Vialard et al. (2012b), i.e. only a few Karcher iterations are required for convergence, and (2) the alternate minimization over *T* and {*R*_*i*_}_1 ≤ *i* ≤ *n*_ provides a faster convergence than the one over *T* with the {*R*_i_} fixed.

Second, we evaluated the empirical convergence of our optimization procedure. Fig. 4a shows the relative distance to the final estimate, i.e.

(23) $$\frac{∥T_{k}-T_{\infty }{∥}_{L_{2}}^{2}}{∥T_{\infty }{∥}_{L_{2}}^{2}},$$

where *T_k_* is the Karcher estimate at iteration *k*, and *T_∞_* is approximated by the last computed estimate. Fig. 4b shows the relative distance between two consecutive estimates, i.e.

(24) $$\frac{∥T_{k+1}-T_{k}{∥}_{L_{2}}^{2}}{∥T_{k}{∥}_{L_{2}}^{2}},$$

with the same notations. On this dataset, we notice that (1) the convergence speeds are coherent with the ones presented in Vialard et al. (2011, 2012b), i.e. only a few Karcher iterations are required for convergence, and (2) the alternate minimization over *T* and {*R*_*i*_}_1 ≤ *i* ≤ *n*_ provides a faster convergence than the one over *T* with the {*R_i_*} fixed.

#### 4.2.4 Transport of initial momenta

To compute the transformations *ϕ^i^* from the screening hippocampi towards the template (Fig. 3), rigid (Ourselin et al., 2001) then non-rigid (Modat et al., 2010) registration algorithms were applied with their default parameters. To check the quality of the registration *ϕ^i^* computed to transport the local descriptor of the patient *i* (first step in 3), the Dice score was computed between the rigidly registered screening image and the template (i.e. *DSC*(*S* ∘ (*R*^*i*^)^− 1^,*T*)) and between the final registered screening image and the template (i.e. *DSC*(*S* ∘ (*ϕ*^*i*^)^− 1^,*T*)).

#### 4.2.5 Computation of the region of interest Ω*_S_*

**Fig. 5:**
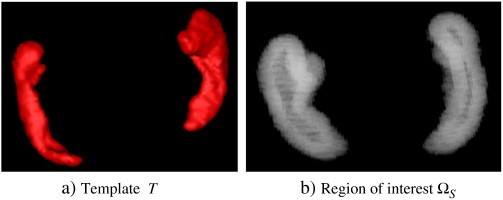
The region of interest Ω*_S_* (visualized with transparency) is designed to select voxels close to the boundary (i.e. close to the surface) of *T*. It is obtained via standard morphological operations, and in this study Ω*_S_* contains 12,531 voxels.

The region of interest Ω*_S_* was restricted around the surface of the template (see Fig. 5), where the high values of the initial momenta lie. Moreover, this allows greater differences of coefficient values from one side to the other when using Sobolev regularization.

More specifically, given a binary template *T* : *Ω* ⊂ ℝ^3^ → [0,1] and a spherical structural element *E_r_* of radius *r* ∈ ℝ defined as

(25) $$E_{r}\overset{\mathrm{def}.}{=}\left\{\left(\omega_{1},\omega_{2},\omega_{3}\right)\in {\mathbb{R}}^{3};\;\omega_{1}^{2}+\omega_{2}^{2}+\omega_{3}^{2}\leq r^{2}\right\},$$

we define the region of interest Ω*_S_* as

(26) $$\Omega_{S}\overset{\mathrm{def}.}{=}\mathrm{Dila}\left(T,E_{r}\right)-\mathrm{Ero}\left(T,E_{r}\right),$$

where Dila and Ero are the standard dilatation and erosion morphological operators. In this study, using *r* = 5, the ROI Ω*_S_* contained 12,531 voxels.

#### 4.2.6 Optimization of the logistic regression model

In the training procedure, we have *n* = 103 observations (one for each patient). As initial momenta are scalar fields in space, each initial momenta has the same dimension as the number of voxels, so *p* = 12,531. Since stable and progressive classes in the dataset are unbalanced, the weighted version of the loss function defined in Section 3.2.3 was used. Solution of the optimization problems was computed via HANSO^4^ with a maximum of 20 iterations.

#### 4.2.7 Performance evaluation

First, the effect of spatial regularizations was compared. The spatial regularizations introduced in Section 3.2 aim at enforcing local correlations between the coefficients in **w**. Using the whole dataset, the effects of the various regularizations were compared. Second, the model was evaluated in terms of classification of AD progression. All patients were classified using a leave-10%-out scheme. From the numbers of true/false positives/negatives (TP, FP, TN, FN), four indicators were used to measure classification accuracy: specificity $\mathrm{Spec}\overset{\mathrm{def}.}{=}\frac{\mathrm{TN}}{\mathrm{TN}+\mathrm{FP}}$, sensitivity $\mathrm{Sens}\overset{\mathrm{def}.}{=}\frac{\mathrm{TP}}{\mathrm{TP}+\mathrm{FN}}$, negative predictive value $\mathrm{NPV}\overset{\mathrm{def}.}{=}\frac{\mathrm{TN}}{\mathrm{TN}+\mathrm{FN}}$, and positive predictive value $\mathrm{PPV}\overset{\mathrm{def}.}{=}\frac{\mathrm{TP}}{\mathrm{TP}+\mathrm{FP}}$. Statistical tests were also performed to evaluate the significance of the differences. Using *N* = 50 random re-orderings of the patients, the Spec + Sens variable was computed 50 times for each regularization and two-sample t-tests were performed.

### 4.3 Effect of spatial regularizations

**Fig. 6:**
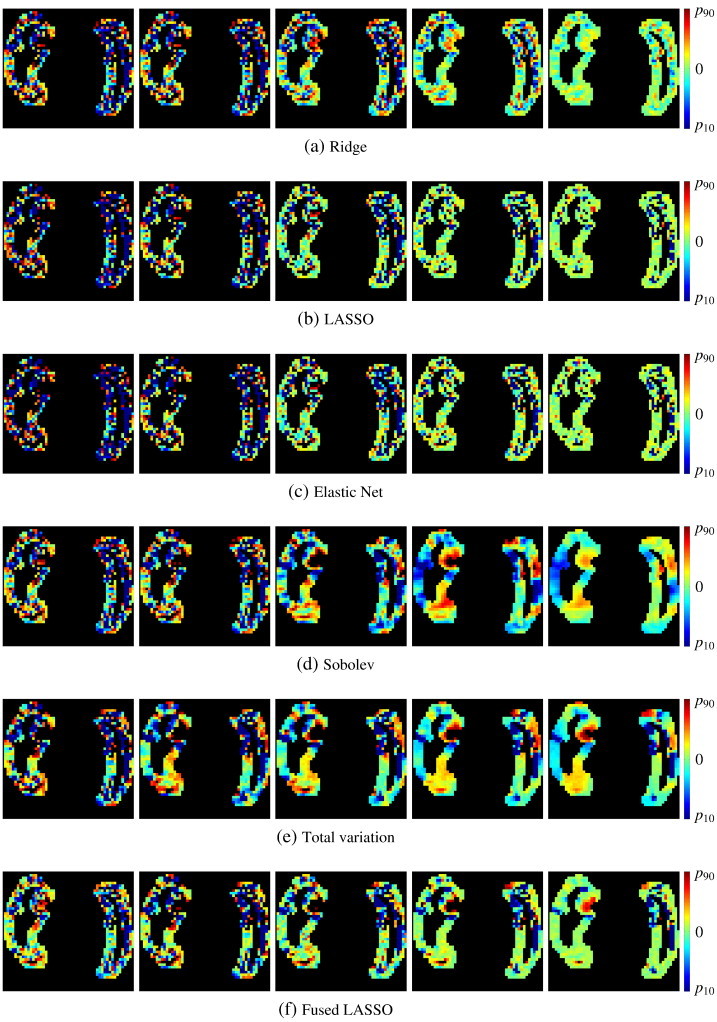
Effects of various regularizations on the solution $\hat{w}$ of the optimization problem. Each small image represents the coefficients of one 2D slice of $\hat{w}$, which is a 3D volume. Zero coefficients are displayed in light green, higher values are going red and lower values are going blue. On each row, the regularization is increasing from left to right, and the 10th and 90th percentiles of the coefficients (resp. *P*_10_ and *P*_90_) correspond to the saturation limits of the colorbar. Panels a, b and c show standard regularizations whereas Panels d, e and f show spatial regularizations. Spatial regularizations provide more structured coefficients.

When using standard regularizations, increasing the regularization does not lead to any spatial coherence (Fig. 6a, b and c). It is interesting to remark that LASSO regularization emphasizes a more limited number of points than ridge regularization. This is particularly clear in the right columns of Fig. 6, where the regularization energy (λJ(**w**) in Eq. (16)) has a significant weight in the total energy. As expected, ElasticNet also gives results which are in-between those of LASSO and those of ridge. In contrast to these regularization techniques, the higher the spatial regularizations, the more structured are the coefficients. Note that delimited areas are coherent across different spatial regularizations. Sobolev regularization leads to smooth coefficient maps (Fig. 6d) whereas total variation tends to piecewise constant maps (Fig. 6e). Finally, fused LASSO adds sparsity by zeroing out the lowest coefficients (Fig. 6f).

#### 4.3.1 Another benefit of spatial regularizations

As mentioned in the Introduction, a motivation to regularize the learning problem is the low number of observations compared to the dimensionality of the problem. However, we can infer another benefit of the use of spatial regularizations. Indeed, to build voxel-based statistical models from the observations of several patients, one needs to align these observations properly. Even though we checked the quality of the alignment to the template, such alignment is not perfect. Adding spatial regularizations in the model is a way to limit the effects of the alignment errors.

### 4.4 Classification of Alzheimer's disease progression

Besides providing a map of coefficients indicating the importance of each voxel with regard to the disease progression, the model presented in this paper can be used to classify the disease progression of new patients. Table 1 displays the classification performance indicators of binary classification using logistic loss and various regularizations.

Without any regularization, the resulting classifier always predicts the same class. Before going any further, let us comment on this point. If all testing subjects are classified in the same class, it means that all the testing points are on the same side on the hyperplane found in the optimization process. Here, unbalanced observations and the chosen optimization strategy are the causes of this result. In the model used, the bias *b* plays a special role and several strategies can be considered, such as 1) optimizing **w** and *b* at the same time, 2) optimizing **w** and *b*, then freezing **w** and optimizing *b*, 3) optimizing **w** and *b*, then freezing **w** and setting *b* using heuristic rules (e.g. setting it to have the same ratio between classes in training and test sets), 4) optimizing **w** with *b* frozen to zero, then optimizing *b*, 5) optimizing **w** with *b* frozen to zero, then setting b using heuristic rules, etc. In initial tests, we realized that some strategies would classify all patients to positive whereas other would classify them all to negative. This happened when the optimization is not regularized. However, this instability with regard to the optimization strategy fades out when the problem is regularized. These initial tests further motivated the use of regularization. Let us note that the above strategy 1) was used in all the results presented in this paper.

All regularizations improve significantly the classification performance, the top 3 being the three spatial regularizations. On this dataset, fused LASSO is the one providing the best results (Spec + Sens = 1.32), closely followed by total variation (Spec + Sens = 1.31).

#### 4.4.1 Comparison with the literature

Using spatial regularizations such as total variation and fused-LASSO, our experiments provide higher performances than the best one reported in Fiot et al. (2012) (Spec + Sens = 1.27). Moreover, the linear classification model used in this paper is simpler than the non-linear SVM used in Fiot et al. (2012). SVM is a very powerful approach, which has been widely studied and successfully used. Many implementations are available, but it can get difficult to modify them and, for example, add spatial regularizations. Besides, only linear SVM can provide an interpretable map of coefficients, but not the non-linear version used in Fiot et al. (2012). On the other hand, a model as simple as the logistic regression can be easily implemented and modified.

### 4.5 Statistical tests

To evaluate the significance of the performance differences found in Table 1, we performed two-sample t-tests. The variable considered was Spec + Sens, and 50 realizations of the variable from random re-ordering of the patients were obtained for each sample. Two regularizations can be considered statistically significantly different if the test has a *p*-value *p* < α = 10^− 3^. These results are presented in Table 2. First, we notice that all regularizations are statistically better than the absence of regularization. Then we notice that all spatial regularizations are statistically better than standard regularizations. Finally, we notice that despite higher prediction accuracy, Elastic Net is not statistically significantly better than ridge in our tests. Similarly, fused-LASSO is not statistically significantly better than total variation in our tests.

### 4.6 Computation time

The various algorithms were implemented in a mix of C++, MATLAB^®^, mex and python. Table 3 reports approximate running time on a standard laptop (Intel^®^ Core™ i7-2720QM CPU at 2.20 GHz, 8 GB of RAM). The geodesic shooting step is linear with the number of patients. The computation of the template is linear with the number of patients and the number of Karcher iterations. One should note that Karcher iterations can have decreasing number of gradient descent iterations, which decreases the total computation time. Then the transport is also linear with the number of patients. So far, it is interesting to notice that all the steps can be easily be divided into different jobs to take advantage of multi-core or distributed architectures. Finally come the learning and classification. The computation time of this step can vary dramatically depending on several parameters such as the training/testing splitting scheme, the optimization algorithm, and the number of regularization parameters to test. In particular, for this exploratory study, we used mainly HANSO algorithm, since the convergence rate of the proximal algorithms mentioned in Section 3.2.2 was too low.

### 4.7 Comparison with the literature

As mentioned earlier, the main contribution of this paper is the comparison of the effects of various regularizations on the solution of binary classification problem with a logistic loss. In the context of longitudinal Alzheimer's disease study, we saw that the use of spatial regularizations techniques was not only leading to better classification results than standard regularizations, but also providing maps of coefficients with improved spatial coherence.

In the literature, a large number of methods are also trying to identify the hippocampal sub-areas that are related to either the conversion of patients to the disease or to other symptoms such as cognitive or memory measures. For example, one can cite Fig. 5 of Frisoni et al. (2008), Fig. 7 of Gutman et al. (2009), Fig. 1 to 5 of Apostolova et al. (2010), and Fig. 3 and 4 of Shen et al. (2012).

Several strategies can be considered to compare the most significant regions found by various methods. One strategy is to transport relevance maps from different methods to the same space. However, transporting information is delicate (Fiot et al., 2012), and one needs to be cautious with such strategy. This transport could be avoided by using the same template for all methods, though this is likely to cause problems if the population studied is not the same. Another strategy is to rank the hippocampal subareas, as it is done for example in Table 2 of Frisoni et al. (2008), and compare the rankings. This strategy would require us to align a map of known hippocampal subareas to our template, and design a ranking algorithm (for example based on $\int_{\omega \in \Omega_{R}}\hat{w}{\left(\omega \right)}^{2}\mathrm{d\omega}$, where Ω*_R_* is a hippocampal subregion).

Comparing qualitatively or quantitatively the subregions that are the most significant with regard to disease progression is out of the scope of this paper. Nonetheless, it is a very interesting perspective, and several strategies including the ones mentioned above are considered for future work.

## 5 Conclusion

In this paper, we studied deformation models for longitudinal population analysis, regularizations and machine learning strategies. In particular, we investigated the combined use of the LDDMM framework and classification with logistic loss and spatial regularizations in the context of Alzheimer's disease. Results indicate that initial momenta of hippocampus deformations are able to capture information relevant to the progression of the disease.

Another contribution of this paper is the joint use of a simple linear classifier with complex spatial regularizations. Achieving results higher than the ones reported in Fiot et al. (2012), which uses non-linear SVM classifier, our method provides in addition coefficient maps with direct anatomical interpretation.

Moreover, we compared Sobolev, total variation and fused LASSO regularizations. While they all successfully enforce different priors (respectively smooth, piecewise constant and sparse), their resulting coefficient maps are coherent one to the other. They improve coefficient maps and their classification performances are statistically better than the ones obtained with standard regularizations.

Now the ideas and results presented in this paper open a wide range of perspectives. First, the question of the representation of patients from images, and in particular the representation of their evolutions for longitudinal population studies was raised. We have used initial momenta encoding the patient evolution in 3D volumes. An interesting research direction is the adaptation of our pipeline to surface representation of shape evolution. Indeed, as we saw in the application studied in this paper, the strong values of the initial momenta lie on the hippocampus volume boundary, in other words on the surface. Second, the question of how to compare evolutions of different patients was raised. We studied the use of Karcher mean and the importance of the regularizations. Even though diffeomorphic deformation models such as LDDMM can provide smooth deformation fields and encode the shape deformation of a patient in a smooth representation, we saw that it is important to regularize spatially across the population (i.e. between patients) in order to be able to build meaningful statistical models for classification and biomarker discovery. On that point, the logistic regression model has proven to be efficient as it can be combined with complex regularizations. Our spatial regularizations gave the best results on our dataset, and another research direction is the study of other regularizations such as group sparsity. Third, another great perspective of this work consists in studying evolutions of patients with more than two time points. In this context, the design of spatio-temporal regularizations (for example in the context of geodesic regression (Niethammer et al., 2011)) is an exciting research direction.

## Figures and Tables

**Table 1:**
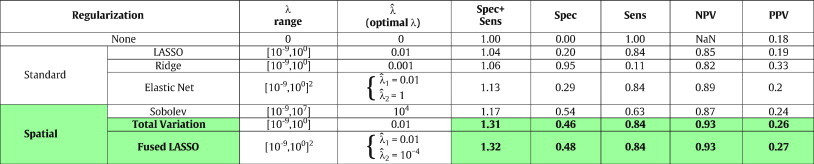
Prediction accuracy of MCI patients' progression.

**Table 2:**
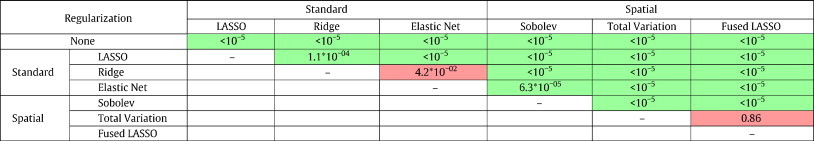
Statistical *p*-values of two-sample t-tests between different regularizations. The variable considered is Spec + Sens, and 50 realizations of the variable from random re-orderings of the patients were obtained for each sample. Two regularizations can be considered statistically significantly different if the test has a *p*-value light green *p* < α = 10^− 3^ (marked in green, red otherwise).

**Table 3:** Computation time of the various steps. (^⁎^): can differ by several orders of magnitude, see Section 4.6 for details.

**Step**	**Computation time**
Preprocessing	A few hours
Geodesic shooting	≈ 1 day
Template computation	≈ 3 days
Transport	≈ 1 day
Learning and classification	From 1 min to several days^⁎^

## Footnotes

3 http://sourceforge.net/projects/utilzreg/.

4 http://www.cs.nyu.edu/overton/software/hanso.
